# The role of the oral microbiome in smoking-related cardiovascular risk: a review of the literature exploring mechanisms and pathways

**DOI:** 10.1186/s12967-022-03785-x

**Published:** 2022-12-12

**Authors:** Katherine A. Maki, Sukirth M. Ganesan, Brianna Meeks, Nicole Farmer, Narjis Kazmi, Jennifer J. Barb, Paule V. Joseph, Gwenyth R. Wallen

**Affiliations:** 1 Translational Biobehavioral and Health Disparities Branch, National Institutes of Health, Clinical Center, 10 Center Drive, Building 10, Bethesda, MD 20814 USA; 2 Department of Periodontics, The University of Iowa College of Dentistry and Dental Clinics, 801 Newton Rd., Iowa City, IA 52242 USA; 3 University of Maryland, School of Social Work, Baltimore, MD USA; 4 National Institute on Alcohol Abuse and Alcoholism, National Institutes of Health, Bethesda, MD USA; 5 National Institute of Nursing Research, National Institutes of Health, Bethesda, MD USA

**Keywords:** Oral microbiome, Microbiota, Oral health, Genetics, Smoking, Cardiovascular risk, Cardiovascular disease

## Abstract

Cardiovascular disease is a leading cause of morbidity and mortality. Oral health is associated with smoking and cardiovascular outcomes, but there are gaps in knowledge of many mechanisms connecting smoking to cardiovascular risk. Therefore, the aim of this review is to synthesize literature on smoking and the oral microbiome, and smoking and cardiovascular risk/disease, respectively. A secondary aim is to identify common associations between the oral microbiome and cardiovascular risk/disease to smoking, respectively, to identify potential shared oral microbiome-associated mechanisms. We identified several oral bacteria across varying studies that were associated with smoking. *Atopobium*, *Gemella*, *Megasphaera*, *Mycoplasma*, *Porphyromonas*, *Prevotella*, *Rothia*, *Treponema*, and *Veillonella* were increased, while *Bergeyella*, *Haemophilus*, *Lautropia*, and *Neisseria* were decreased in the oral microbiome of smokers versus non-smokers. Several bacteria that were increased in the oral microbiome of smokers were also positively associated with cardiovascular outcomes including *Porphyromonas*, *Prevotella*, *Treponema*, and *Veillonella.* We review possible mechanisms that may link the oral microbiome to smoking and cardiovascular risk including inflammation, modulation of amino acids and lipids, and nitric oxide modulation. Our hope is this review will inform future research targeting the microbiome and smoking-related cardiovascular disease so possible microbial targets for cardiovascular risk reduction can be identified.

**Supplementary Information:**

The online version contains supplementary material available at 10.1186/s12967-022-03785-x.

Cardiovascular disease (CVD) greatly increases morbidity and mortality, and contributed to approximately 18.6 million global deaths in 2019 [1]. Data from epidemiological studies show strong links between smoking and CVD, and smoking is directly associated with nearly 20 percent of deaths from CVD [2]. It has been established that smoking is a main modifiable risk factor in developing early atherosclerosis, CVD, and death. Smoking leads to increased systemic inflammation, platelet activation, and dysregulation of vascular smooth muscle control, thus affecting the cardiovascular system [3]. The paired endothelial and vascular dysfunction and platelet/clotting abnormalities create a pro-atherogenic environment in which stable and unstable plaques can develop. The development of atherosclerotic plaque leads to various types of CVD-related morbidity, including myocardial infarction, stroke, and congestive heart failure. Periodontitis, a condition associated with cardiovascular risk and CVD [4–6], occurs at significantly higher rates in subjects who smoke cigarettes [7]. Periodontitis is associated with characteristic bacterial alterations and local inflammatory responses [8], but the documented connection of periodontitis to systemic inflammation suggests local disruptions in oral bacteria may have implications that reach beyond the oral environment [9, 10].

The human microbiome is defined as the collection of bacteria (and their genes) that inhabit and coexist within humans. These bacteria play an integral role in vital physiologic processes such as metabolite production through fermentation of complex carbohydrates, modulation of host immune responses, and regulation of lipid metabolism [11, 12]. Since the development of the Human Microbiome Project, the role of the human microbiome in the maintenance of health and several physiological processes has been of great interest [13], including those associated with cardiovascular health [14]. Several internal and external factors, including diet and smoking, influence the structure and function of these microbial communities [15, 16]. Alteration in bacterial composition of microbiome communities across body sites have been associated with several diseases, including hypertension, obesity, hyperlipidemia and alcohol use disorder [17–20]. There are mechanisms linking the microbiome to CVD, although most of this research focuses on the gut microbiome and researchers understand less about the interaction between bacterial communities of the oral environment and host physiology [20, 21].

The oral cavity is a highly complex, open ecosystem with several microenvironments [22]. Bacterial species colonize the mouth in organized communities called biofilms that are in continual interface with the external environment and host immune system through the oral mucosal epithelium [23]. These biofilms pose advantages to bacteria of the oral microbiome by increasing bacteria-specific nutrient availability and providing protection from environmental or physiologic stressors [24]. Importantly, the structure and composition of oral microbiome biofilms varies across oral sampling sites [25]. Mucosal oral surfaces with high cellular turnover have largely different characteristics versus non-mucosal surfaces (e.g. teeth), despite their close proximity within the oral cavity. Furthermore, the mucosal and non-mucosal microenvironments of the mouth have significant differences in oxygen exposure, pH, and temperature, creating favorable growing conditions for different species of bacteria. Each oral microenvironment, i.e., the subgingival sulcus, tongue dorsum, buccal mucosa and saliva, has distinct but temporally stable bacterial populations that may have disparate responses to smoking exposure.

Importantly, the oral cavity is exposed to different chemicals and substances when e-cigarette vapor versus tobacco smoke is inhaled. For example, cigarette smoking exposes the oral environment to irritants, chemicals, and carcinogens through incomplete combustion byproducts, including polycyclic aromatic hydrocarbons and nitrosamine 4-(methylnitrosamino)-1-(3-pyridyl)-1-butanone, along with carbon monoxide [26]. Electronic-cigarette (e-cigarette) use, originally proposed as a safer alternative to cigarette smoking, results in oral exposure to nicotine dissolved in solvents including alcohols, glycerin, propylene glycol, and propylene oxide through the inhalation of aerosols [27]. In e-cigarettes, levels of volatile organic compounds, nitrosamines, aldehydes, and metals vary considerably across different brands making understanding the link between aerosol inhalation and oral chemical exposure difficult [28]. Both cigarette and e-cigarette smoking have been shown to alter the oral environment by decreasing saliva volumes, increasing saliva viscosity, destroying protective salivary macromolecules and triggering inflammatory responses and chronic inflammation [29–31].

Potential mechanisms between oral microbes, smoking, and cardiovascular conditions are largely unexplored. The physiologic implications of many of the chemicals present in cigarette smoke are historically understudied, and even less is known about how the oral microbiome may modulate interactions between cigarette smoke versus e-cigarette vapor and human physiology. The circulatory system provides a route where the bacteria of the oral microbiome may influence CVD risk. Each tooth has a blood supply, and byproducts of oral bacterial metabolism can produce metabolites or endotoxins that migrate into the bloodstream, causing systemic inflammation that affects other parts of the body [32]. This is a potential mechanism, but the specific pathways linking smoking to CVD through the oral microbiome are still unknown. As the oral cavity and its associated microbial communities are the first contact with inhaled cigarette smoke, responses of the oral microbial taxa to smoke products and subsequent implications for health are important to elucidate. Therefore, this manuscript reviews associations between the oral microbiome and smoking, and the oral microbiome and CVD, focusing on shared associations and potential oral microbial-mediated mechanisms linking smoking to CVD risk.

In collaboration with our institutional biomedical librarian, PubMed was searched for primary literature to yield sources focused on smoking and the oral microbiome, and smoking and cardiovascular risk or disease, respectively. Please see Additional file 1 for search terms, combinations and strategy used in the PubMed searches for primary literature source data acquisition.

## Cigarette smoking and oral microbiome compositional changes

As the oral microbial communities exist in an intricate and coordinated ecosystem, changes in the oral environment result in adaptation and shifts in which bacteria thrive based on their phenotypic and colony characteristics. Several factors are known to modify the oral environment and influence the composition of the oral microbiome as a result. Factors (both internal and external) such as diet, certain medications, smoking, moisture of the environment, and periodontal disease status alter the amount and composition of saliva, which impacts the surrounding epithelial tissues and oral cavity [33]. As previously mentioned, cigarette smoking exposes the oral environment to irritants, chemicals, and carcinogens [26], and alters the oral environment promoting inflammatory responses [29–31]. Since niche-specific colonization exists in the oral cavity, we will review the associations between smoking and the oral microbiome across different sites: whole mouth, saliva, tongue dorsum, supragingival plaque, and subgingival plaque (see Table 1 for a list of studies that evaluated associations between smoking and the oral microbiome). The relationship between smoking and alpha diversity was greatly heterogenous across studies. Alpha diversity is defined as microbial diversity within an individual sample, and is often used as a global indicator of the bacterial characteristics within a microbiome sampling site [22]. Smoking was associated with an increased alpha diversity of whole mouth [34], buccal mucosa [35], and saliva [36, 37] samples in some research, while other studies reported decreased alpha diversity in whole mouth [38], saliva [39, 40] and tongue dorsum [41] samples in smokers versus non-smokers. Additionally, smoking was associated with decreased alpha diversity of subgingival samples of patients with and without chronic periodontal disease [42]. Other groups reported no differences in alpha diversity of whole mouth (via mouthwash) [43], buccal [44], tongue dorsum [45] and saliva [45, 46] samples of smokers, compared to non-smokers.

**Table 1:** Included studies examining the association between smoking and the oral microbiome

Author, year	Oral sample site	Aim	Population studied	Sequencing	Region if 16S	Citation
Cigarette smokers						
Al-Zyoud et al., 2019	Saliva	Investigate the shift in the salivary microbiota between smokers and non-smokers in Jordan	Nonsmoking subjects (n = 51), subjects who smoke (n = 49), total sample (n = 100)	16S	V3–V4	[37]
Beghini et al., 2019	Oral rinse	To evaluate the effect of tobacco exposure on the oral microbiome from oral rinse samples in the 2013–14 New York City Health and Nutrition Examination Study	Total sample (n = 259)	16S	V4	[43]
Coretti et al., 2017	Subgingival	Assess the subgingival microbiota in smoker patients with chronic periodontitis, non‑smoker patients with chronic periodontitis and healthy controls	Subjects with chronic periodontitis who smoke (n = 6), non‑smoker patients with chronic periodontitis (n = 6), nonsmoking subjects without periodontitis (n = 8), total sample (n = 20)	16S	V3–V4	[42]
Duan et al., 2017	Saliva	Studied the impact of smoking on the salivary microbiome and its further influence on marginal bone loss around an implant during a 3-month bone-healing period	Smokers (n = 10) and non-smokers (n = 10) presenting for single-tooth replacement, total sample (n = 20)	16S	V4	[39]
Gaetti-Jardim, 2018	Supra- and sub-gingival plaque	Aimed to evaluate the effects of conventional radiotherapy on the prevalence and populations of oral microorganisms in head and neck cancer patients who did not receive adequate preventive dental care	Subjects with head and neck cancer (n = 28)	Culture dependent	N/A	[56]
Ganesan et al., 2017	Subgingival plaque	Analyzed 16S sequences from non-smoking normoglycemic individuals (controls), smokers, diabetics and diabetic smokers with periodontitis, as well as periodontally healthy controls, smokers and diabetics to assess subgingival bacterial biodiversity and co-occurrence patterns	Normoglycemic non-smokers with periodontitis (n = 14), hyperglycemic non-smokers with periodontitis (n = 9), normoglycemic smokers with periodontitis (n = 16), and hyperglycemic smokers with periodontitis (n = 8), normoglycemic non-smokers without periodontitis (n = 14), hyperglycemic non-smokers without periodontitis (n = 12), normoglycemic smokers without periodontitis (n = 12), total sample (n = 175)	16S	V1–V3; V7–V9	[58]
Gopinath et al., 2022	Buccal swab	Investigate the compositional and functional attributes of the oral bacteriome of smokeless tobacco users and smokers relative to controls by 16S rRNA metagenomic sequencing in an Indian population	Smokers (n = 17), smokeless tobacco users (n = 14), age-matched non-smokers (n = 13), total sample (n = 44)	16S	V3–V4	[35]
Hsiao et al., 2018	Saliva	Investigated the association between oral bacterial profile and oral squamous cell carcinoma risk in a case–control study	Subjects with oral squamous cell carcinoma (n = 138), controls (n = 151), total sample (n = 289)	16S	V3–V5	[54]
Jia et al., 2021	Saliva	Improve our understanding of the impact of cigarette smoking on the oral microbiota in the Chinese population	Subjects from Guangdong Providence (n = 150), subjects from Yangquan city (n = 81), subjects from Mishan city (n = 85), total sample (n = 316)	16S	V4	[36]
Karabudak et al., 2019	Buccal swab	Investigate the effect of smoking on the buccal microbiome and to analyze the descriptive ability of each of the seven hypervariable regions in their 16S rRNA genes	Smokers (n = 20), non-smokers (n = 20), total sample (n = 40)	16S	V2, V3, V4, V6–7, V8, V9	[44]
Karasneh et al., 2017	Subgingival plaque	Investigate the impact of smoking on the subgingival bacterial profile in both healthy adults and chronic periodontitis patients	Subjects with chronic periodontitis (n = 37 non-smokers and n = 18 smokers), subjects without periodontitis (37 non-smokers and 18 smokers), total sample (n = 94)	16S	V1–V9	[59]
Lin et al., 2019	Saliva	Leveraged next generation sequencing for microbiome and functional neuroimaging to enable the delineation of microbiome-brain network links as well as their relationship to cigarette smoking	Smokers (n = 30), non-smokers (n = 30), total sample (n = 60)	16S	V4	[52]
Mukherjee et al., 2018	Oral rinse	Assessed the relationship of microbial dysbiosis with smoking and markers of human immunodeficiency virus disease	HIV-infected smokers (n = 48), HIV-infected non-smokers (n = 24), HIV-uninfected smokers (n = 24), total sample (n = 96)	16S	V4	[50]
Murugesan et al., 2020	Saliva	Characterize the salivary microbiome composition in the Qatari population, and to explore specific microbial signatures that can be associated with various lifestyles and different oral conditions	Total sample (n = 997)	16S	V1–V3	[40]
Pushalkar et al., 2020*	Saliva	Evaluate the effects of e-cigarette aerosol and its influence on human salivary microbiome and immune health. Additionally, the authors evaluate the influence of e-cigarette aerosols on infection efficiency of oral pathogens in pre-cancerous and cancer cell lines using a novel e-cigarette aerosol-generating machine and pro-inflammatory immune mediators	Smokers (n = 40), never smokers (n = 39), e-cigarette users (n = 40), total sample (n = 119)	16S	V3–V4	[46]
Renson et al., 2019	Oral rinse	Describe sociodemographic variation of oral microbiomes in a subsample of the 2013–14 population-based New York City Health and Nutrition Examination Study	Total sample (n = 282)	16S	V4	[47]
Rodríguez- Rabassa et al., 2018	Saliva	Investigated the effects of cigarette smoking on bacterial diversity and host responses compared to non-smokers	Non-smokers (n = 16), current smokers (n = 18), total sample (n = 34)	16S	V3–V4	[51]
Sato et al., 2020a	Tongue dorsum	Investigated the bacterial species composition in the tongue microbiome, as well as single-nucleotide variant profiles and gene content of these species, in never and current smokers by utilizing metagenomic sequences	Never smokers (n = 234), current smokers (n = 52), total sample (n = 286)	Shotgun metagenomic sequencing	N/A	[55]
Sato et al., 2020b	Tongue dorsum	Used 16S rRNA amplicon sequencing of tongue-coating samples obtained from East Asian subjects who were current, former, or never smokers to identify differences in their tongue microbiomes and related metagenomic functions	Never smokers (n = 384), former smokers (n = 129), current smokers (n = 144), total sample (n = 657)	16S	V3–V4	[41]
Shay et al., 2020	Oral rinse	Characterize the bacteriome, mycobiome and mycobiome-bacteriome interactions of oral wash samples in head and neck squamous cell carcinoma patients and to determine if they are distinct from those of the oral wash of matched non-head and neck squamous cell carcinoma patients	Subjects with head and neck squamous cell carcinoma (n = 46), subjects without cancer (n = 46), total sample (n = 92)	16S	V1–V2	[49]
Suzuki et al., 2022	Saliva and tongue dorsum	Investigated the differences in the microbial composition of the tongue directly exposed to cigarette smoke in smokers with that of nonsmokers	Saliva (n = 47) and tongue dorsum (n = 50) samples of healthy volunteers, total sample (n = 50)	16S	V3–V4	[45]
Thomas et al., 2014	Oral biofilm/whole mouth swab	Investigate the effects of the chronic use of alcohol and tobacco over the oral microbiome, in terms of diversity and composition, using 16S rRNA gene sequencing	Subjects with no alcohol or tobacco consumption (n = 9); subjects with heavy alcohol and tobacco consumption (n = 7), subjects who smoke but do not consume alcohol (n = 6), total sample (n = 22)	16S	V1	[38]
Vallès et al., 2018	Oral rinse	Compared the effects of cigarette, dokha and shisha use on community composition of the oral microbiome by high-throughput sequencing of the bacterial 16S rRNA gene in 330 participants from the “UAE Healthy Future” pilot study	Subjects who smoke (n = 105), subjects who do not smoke (n = 225), total sample (n = 330)	16S	V4	[34]
Wolff et al., 2019	Supragingival plaque	Study patterns in pathogenic biofilm composition to characterize the oral microbiome present in tooth surfaces with and without caries. Smoking and socio-economic status were studied as exploratory variables	Total sample (n = 56)	16S	V4	[57]
Yeo et al., 2019	Saliva	Address the gap in knowledge by reporting on the anthropometrics and cardiometabolic health of a resettled Temiar community and investigated their saliva microbiome in association with their health	Total sample (n = 72)	16S	V3–V4	[53]
Yeoh et al., 2019	Oral rinse	Collected oral rinse samples from patients showing symptoms of acute tonsillitis and compared their oral cavity microbial community composition to healthy individuals without oral disease	Healthy (n = 165), tonsillitis (n = 43), total sample (n = 208)	16S	V3–V4	[48]
E-cigarette smokers						
Ganesan et al., 2020	Subgingival plaque	Investigate the effects of e-cigarettes on the subgingival microbiome using complementary approaches to achieve comprehensive insights into community assembly, dynamics, and function, as well as the impact of this community on the host’s immunoinflammatory response	Smokers (n = 25), non-smokers (n = 25), e-cigarette users (n = 20), former smokers currently using e-cigarettes (n = 25), concomitant cigarette and e-cigarette users (n = 28), total sample (n = 123)	Shotgun metagenomic sequencing	N/A	[15]
Pushalkar, 2020*	Saliva	Study the in vivo effects of e-cigarette aerosol and its influence Additionally, the authors evaluated the influence of e-cigarette aerosols on infection efficiency of oral pathogens in pre-cancerous and cancer cell lines using a novel e-cigarette aerosol-generating machine and pro-inflammatory immune mediators	Smokers (n = 40), never smokers (n = 39), e-cigarette users (n = 40), total sample (n = 119)	16S	V3–V4	[46]

*16S* 16S ribosomal ribonucleic acid sequencing, *HIV* human immunodeficiency virus, *V* variable region of the 16S ribosomal ribonucleic acid gene

*Denotes study included comparisons with both cigarette and e-cigarette smokers

### Whole mouth, saliva and buccal mucosa

Researchers collected whole mouth samples by swabbing multiple surfaces of the mouth [38] or collecting an alcohol- [43, 47–50] or saline-based [34] mouthwash to analyze microbial populations across multiple oral microenvironments. Similar to whole mouth samples, saliva microbiome composition may be influenced by other oral microbial niches, but sample collection occurred through spontaneous passive [36, 37, 39, 40, 45, 51–54] or stimulated [46] salivary fluid collection in the reviewed studies. In saliva or buccal mucosa samples of cigarette smokers, the majority of studies demonstrated an increased relative abundance (RA) of *Actinomyces* [36, 37, 48], *Actinomyces* species [46, 52], *Aggregatibacter* [53], *Bacteroides* [52], and *Lachnoanaerobaculum* [39] versus non-smokers. Cigarette smoking also increased the RA of *Alloprevotella* species [39], *Campylobacter* [36, 43, 53], *Dialister* [34], *Eubacterium* [52] and *Eubacterium* species [39, 52] in saliva and whole mouth samples versus subjects who did not smoke. Conversely, several studies reported a decreased RA of *Acinetobacter* species [39], *Bifidobacterium* [47], *Catonella* [39], *Capnocytophaga* [40, 45], *Cardiobacterium* [36, 45], *Granulicatella* [38, 48, 51], *Lactococcus* [40, 47], *Leptotrichia* [34, 48, 49, 51], *Pseudomonas orientalis* [39], *Selenomonas* [39, 52], and *Selenomonas* species [39] in saliva and whole mouth samples of cigarette smokers versus non-smokers. The RA of *Actinobacillus* had different associations with cigarette smoking, where the RA of *Actinobacillus* was increased in saliva samples [51] but decreased in whole mouth samples [34, 47] in cigarette smokers (versus non-smokers). There were also conflicting results on the influence of cigarette smoking on the RA of *Fusobacterium* and *Fusobacterium* species in the whole mouth, buccal mucosa and saliva samples. For example, several studies reported both an increased RA of genus-level *Fusobacterium* and *Fusobacterium* species (specifically *F. nucleatum*; [35, 46, 48, 52, 54]) and decreased RA of *Fusobacterium* and *Fusobacterium* species (specifically *F. periodonticum*; [34, 37, 46, 49, 51]) in cigarette smokers versus non-smokers (see Additional file 2: Table S1 for all smoking-associated oral bacteria responses in the reviewed studies).

### Tongue dorsum

Two studies by the same group [41, 55] explored the impact of smoking on the oral microbiome using tongue dorsum samples. These studies reported that cigarette smoking was associated with a decrease in the RA of *Alloprevotella*, *Campylobacter*, *Cardiobacterium*, *Capnocytophaga*, *Fusobacterium*, *Eubacterium*, and *Lachnoanaerobaculum* versus non-smoking subjects [41]. Two independent groups reported that smoking was associated with a decrease in *Peptostreptococcus* and *Catonella* in tongue dorsum samples, compared to non-smokers [41, 45]. Cigarette smoking was also associated with an increased RA of species-level *Eubacterium brachy*, *Eubacterium nodatum*, *Eubacterium saphenum*, *Filifactor alocis*, *Fusobacterium nucleatum*, and *Mogibacterium timidum* in tongue dorsum samples compared to non-smokers [41, 55].

### Supragingival and subgingival plaque

Supragingival and subgingival plaque samples were collected by soft tissue removal [42] or by biofilm [56, 57] and direct plaque [58, 59] collection using sterilized paper points in patients with and without periodontal disease, depending on the study design (Table 1). Cigarette smoking was associated with an increased RA of *Bifidobacterium* [42, 58] and *Campylobacter rectus* [59], *Eikenella corrodens* [59], *Fusobacterium* [42], *Granulicatella* [42], and *Selenomonas sputigena* [59] in subgingival plaque samples and an increased RA of *Haemophilus parainfluenzae* [57] in supragingival samples versus non-smoking subjects. Conversely, the RA of *Capnocytophaga ochracea* [59] and *Pseudomonas* [42] in subgingival plaque samples were decreased in cigarette smokers versus non-smokers.

### Similar bacterial associations across multiple sites

**Fig. 1:**
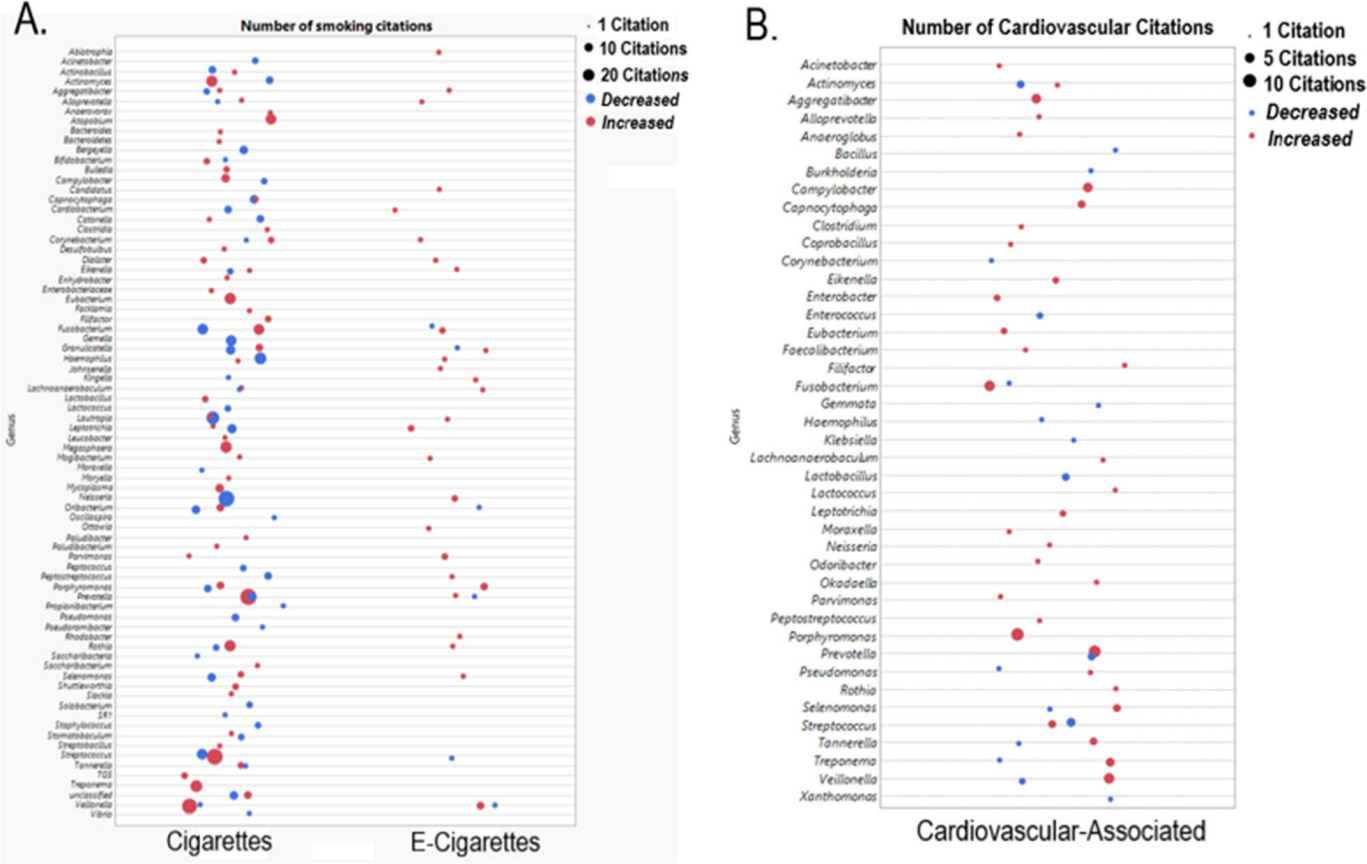
A sub-section of the genera that were cited in manuscripts that studied associations of oral bacteria with cigarette smoking, electronic cigarette smoking, and cardiovascular risk/disease, respectively. Red circles indicate a significant increase of the corresponding oral microbiome taxon to cigarette smoking, e-cigarette smoking or cardiovascular variables, while blue indicate a significantly decreased relative abundance. These figures were generated in JMP™ Version 14 Data Discovery Statistical Software. Data is shown across all oral sites and larger circles indicate more references to an increase or decrease in the specified genus. Species-level features were collapsed to the genus level for illustrative purposes. Taxa at the species and genus level used to make this figure, and the corresponding citations, can be found in Additional file 2: Table S1 (oral microbiome and smoking) and Additional file 3: Table S2 (oral microbiome and cardiovascular risk/disease). *E-cigarette* electronic cigarette

Specific bacterial taxa had similar associations with cigarette smoking in multiple oral microbiome sampling sites (i.e. a shared increase or decrease in RA in response to cigarette smoking in more than one oral microbiome site; Table 2 and Fig. 1A). For example, cigarette smoking was associated with an increased RA of *Atopobium* and *Atopobium* species [34, 36, 37, 41, 45, 55, 58], *Rothia* and *Rothia* species [36, 38, 41, 44, 46, 52, 55], *Lactobacillus* [50, 58], *Mycoplasma* and *Mycoplasma hyosynoviae* [34, 41, 52], *Megasphaera* and *Megasphaera micronuciformis* [34, 36, 37, 39, 41, 43, 46, 55], *Tannerella* and *Tannerella forsythia* [39, 41, 52], *Corynebacterium* and *Corynebacterium* species [41, 57], *Prevotella* and *Prevotella* species [34, 36–40, 44, 46, 52, 55], *Streptococcus* and *Streptococcus* species [41, 44, 55, 58], *Porphyromonas* and *Porphyromonas* species [34, 46, 55], *Treponema* and *Treponema* species [34, 42, 45, 48, 52, 55, 59], and *Veillonella* or *Veillonella* species [36–38, 43, 44, 46, 48, 50, 55, 58], compared to non-smokers. Interestingly, the RA of *Lactobacillus* and *Rothia* were also increased in subjects exposed to secondhand smoke [47]. Cigarette smoking was associated with a decreased RA of *Gemella* [37, 38, 40, 41, 43, 45, 47], *Haemophilus* and *H. haemolyticus* [34, 37, 41, 43, 45, 47, 55], *Neisseria* and *Neisseria* species [34, 36–38, 41, 43, 47, 50, 52, 53, 55, 58], *Bergeyella* [37, 43, 47, 48], *Oribacterium* and *Oribacterium* species [39, 41, 46, 52], *Lautropia* and *L. mirabilis* [34, 36, 37, 41–43, 47, 52, 55, 58] in most oral sites versus subjects who did not smoke. An exception to the previous inverse association between bacterial RA and cigarette or e-cigarette smoking was an increased RA of *L. mirabilis* in supragingival [57] samples and increased *Oribacterium* in the whole mouth [48] and subgingival plaque [42] samples in smokers compared to non-smokers. Additionally, in cigarette smokers (versus non-smokers), the RA of *Actinobacillus* was increased in saliva samples [51] but decreased in whole mouth samples [34, 47]. Not all taxa had agreement across studies when reporting the association between RA and cigarette smoking. For example, in the whole mouth and saliva samples, there were almost an equal number of studies reporting a decreased [40, 43, 49, 51] and increased [37–39, 46, 49] RA of *Streptococcus* or *Streptococcus* species, and the association between smoking and *Prevotella* RA was equivocal in subgingival plaque samples, with one study reporting an increase [58] and the other a decrease [59] in RA versus non-smoking subjects. Current cigarette use was associated with a decreased RA of *Bergeyella*, *Haemophilus*, *Lautropia*, and *Neisseria* compared to former smokers in whole mouth samples [47], indicating that abstinence from smoking might reverse the smoking-associated decrease in RA of these taxa.

**Table 2:** Bacteria of the oral microbiome with similar responses to smoking across multiple sites

Bacteria	Whole mouth	Saliva	Tongue dorsum	Subgingival plaque^a^	Buccal	References
*Atopobium* and *Atopobium* species	↑	↑	↑	↑		[34, 36, 37, 41, 45, 55, 58]
*Bergeyella*	↓	↓				[37, 43, 47, 48]
*Corynebacterium* and *Corynebacterium* species			↑	↑		[41, 57]
*Gemella*	↑	↑	↑			[37, 38, 40, 41, 43, 45, 47]
*Haemophilus* and *H. haemolyticus*	↓	↓	↓			[34, 37, 41, 43, 45, 47, 55]
*Lactobacillus*	↑			↑		[50, 58]
*Lautropia* and *Lautropia mirabilis*	↓	↓	↓	↓↑^b^		[34, 36, 37, 41–43, 47, 52, 55, 57, 58]
*Megasphaera* and *Megasphaera micronuciformis*	↑	↑	↑			[34, 36, 37, 39, 41, 43, 46, 55]
*Mycoplasma* and *Mycoplasma hyosynoviae*	↑	↑	↑			[34, 41, 52]
*Neisseria* and *Neisseria* species	↓	↓	↓	↓		[34, 36–38, 41, 43, 47, 50, 52, 53, 55, 58]
*Oribacterium* and *Oribacterium* species	↑	↓	↓	↑		[39, 41, 42, 46, 48, 52]
*Peptococcus*		↓	↓			[36, 41]
*Porphyromonas* and *Porphyromonas* species	↑	↑	↑			[34, 46, 55]
*Prevotella* and *Prevotella* species	↑	↑	↑	↑↓^b^	↑	[34, 36–40, 44, 46, 52, 55, 58, 59]
*Rothia* and *Rothia* species	↑	↑	↑		↑	[36, 38, 41, 44, 46, 52, 55]
*Streptococcus* and *Streptococcus* species	↑↓^b^	↑↓^b^	↑	↑	↑	[37–41, 43, 44, 46, 49, 51, 55, 58]
*Tannerella* and *Tannerella forsythia*		↑	↑			[39, 41, 52]
*Treponema* and *Treponema* species	↑	↑	↑	↑		[34, 42, 45, 48, 52, 55, 59]
*Veillonella* and *Veillonella* species	↑	↑	↑	↑	↑	[15, 36–38, 43, 44, 46, 48, 50, 55, 58]

^a^Subgingival and/or supragingival plaque

^b^Equal number of studies reporting an increased or decreased relative abundance of bacterial taxa in association with smoking

## Cigarette smoking and oral microbiome-associated metabolite pathway and inflammatory biomarker changes

Glycine, serine, and threonine metabolism pathways, amino acid enzyme and phenylalanine, tyrosine, and tryptophan biosynthesis pathways were upregulated in smokers’ saliva, tongue dorsum and buccal mucosa samples [35, 36, 52]. In studies that documented the presence or absence of periodontal disease, smoking subjects without periodontal disease had higher levels of protein/amino acid biosynthesis and metabolism pathway genes in saliva and buccal mucosa samples compared to non-smokers without periodontal disease [35, 52]. Interestingly, the same pathways (glycine, serine, and threonine metabolism and phenylalanine, tyrosine, and tryptophan biosynthesis pathways) were downregulated in subgingival plaque samples of subjects who smoked and also carried a diagnosis of periodontal disease, suggesting that sampling site and/or associated periodontal disease may influence metabolic responses to smoking [42]. The galactose metabolism pathway was downregulated [55], while lipopolysaccharide biosynthesis pathways were upregulated in tongue dorsum samples of smokers compared to non-smokers [52]. In saliva samples, smoking was associated with a decreased abundance of general lipid metabolism pathways, but increased polyketide metabolism pathways compared to non-smokers [52]. The relationship between smoking and salivary cytokine levels had conflicting results. In one study, saliva levels of pro-inflammatory cytokines tumor necrosis factor (TNF)-α, INF-ɣ, and interleukin (IL)-4 were significantly increased in non-smokers compared to cigarette smokers [46]. In another study, smoking was associated with increased levels of pro-inflammatory IL-2 and IL-4 and decreased levels of anti-inflammatory IL-10 versus non-smokers [51]. Finally, after adjusting for multiple factors, both cigarette smoking and detectible *Porphyromonas gingivalis* bacteria in subgingival plaque samples were associated with plasma high-sensitivity C-reactive protein (CRP) levels [60].

## Electronic-cigarette smoking and oral microbiome compositional changes

The studies reporting associations between the oral microbiome and e-cigarette smoking are limited, but the two studies reported in this review present a comprehensive evaluation of e-cigarette exposure on the saliva and subgingival microbiome communities in addition to the metabolic pathway and cytokine alterations [15, 46]. E-cigarette smoking was associated with an increased RA of *Abiotrophia*, *Aggregatibacter*, *Cardiobacterium*, *Eikenella*, *Granulicatella*, *Haemophilus*, *Johnsenella*, *Kingella*, *Lachnoanaerobaculum*, *Leptotrichia*, *Mogibacterium*, *Parvimonas*, *Peptostreptococcus*, and *Selenomonas* in subgingival plaque samples of e-cigarette smokers versus non-smokers [15]. In saliva samples, there was an increased RA of species-level *Alloprevotella tannerae*, *Corynebacterium durum*, *Dialister invisus*, *Leptotrichia wadei*, *Parvimonas micra*, *Prevotella oris*, and *Veillonella dispar* in e-cigarette smokers versus non-smokers [46].

Conversely, the RA of *Granulicatella adiacens*, *Oribacterium parvum*, *Prevotella* sp.* HMT 317*, *Streptococcus oralis* subsp.* tigurinus clade 071*, and *Veillonella rogosae* were decreased in saliva samples of e-cigarette smokers versus non-smokers [46]. The RA of genus-level *Fusobacterium* and *Neisseria* were also increased in saliva samples compared to both cigarette smokers and non-smokers [46]. *Fusobacterium* species, specifically *F. nucleatum* and *F. periodonticum*, were increased and decreased, respectively, in saliva samples of e-cigarette smokers versus non-smokers [46]. Additionally, *Porphyromonas* species, specifically *P. gingivalis*, *P. endodontalis* and *P. pasteri*, were all increased in saliva samples of e-cigarette smokers versus non-smokers [46].

## Electronic-cigarette smoking and oral microbiome-associated metabolite pathway and inflammatory biomarker changes

Several carbohydrate metabolism pathways were elevated in subgingival plaque samples of e-cigarette users, including the central carbohydrate metabolism, one carbon metabolism, fructose kinase, and the monosaccharide, disaccharide and oligosaccharide metabolism pathways compared to non-smokers [15]. Additionally, in subgingival plaque samples of e-cigarette users, lipid A biosynthesis, glycerol kinase, glycerate kinase lipid metabolism pathways, and general protein/amino acid metabolism genes were all upregulated in e-cigarette users [15]. Lysine fermentation, alanine biosynthesis, and arginine biosynthesis pathways were also upregulated in subgingival plaque samples of e-cigarette users [15]. Lipopolysaccharide biosynthesis pathways were upregulated in both subgingival plaque samples of e-cigarette users and tongue dorsum samples of e-cig smokers compared to non-smokers [15, 52]. In e-cigarette users, general protein/amino acid metabolism genes were upregulated in plaque samples compared to non-smokers [15].

E-cigarette smokers had significantly higher levels of the pro-inflammatory cytokines IL-2, IL-6, TNF-ɑ, and INF-ɣ, and lower levels of the anti-inflammatory cytokine IL-10 present in the gingival crevicular fluid compared to non-smokers [15]. Additionally, gingival crevicular fluid levels of the pro-inflammatory cytokine GM-CSF was higher in e-cigarette users versus cigarette smokers and non-smokers [15]. In another study, salivary levels of the pro-inflammatory cytokine IL-2 were elevated in e-cigarette smokers versus non-smokers [46]. Salivary IL-6 and IL-1ß were also elevated in e-cigarette users versus cigarette smokers and non-smokers in this study, although these differences were not significant [46]. Conversely, the pro-inflammatory cytokine TNF-α had the highest saliva concentrations in non-smokers, followed by cigarette smokers, and finally e-cigarette smokers [46]. Finally, FaDu and Leuk-1 cell lines exposed to e-cigarette aerosol and individually co-infected with the bacterial taxa *P. gingivalis* and *F. nucleatum*, respectively, displayed higher mRNA levels of the pro-inflammatory cytokines IL-1ß, TNF-α, IFN-ɣ, IL-6, and IL-8, when compared to the same co-infected cells exposed to air [46].

## Cardiovascular disease/risk and oral microbiome compositional changes

Alterations in the oral microbiome and clinical periodontitis have been thought to mediate CVD and CVD risk by promoting systemic low-grade inflammation through pathogenic bacteria and their byproducts [61]. Importantly, many current reviews on the link between oral bacteria and CVD have focused on specific bacteria associated with periodontitis [6, 62], and not how changes in other bacterial members of the oral microbiome community may contribute to CVD through cardiovascular migration or endotoxemia. Across most studies analyzed in this review, the number of smokers reported ranged from 11 to 80% in the CVD group (Table 3).

**Table 3:** Included studies examining the association between cardiovascular risk/disease and the oral microbiome

Author, year	Oral sample site(s)	Aim	Population studied	% Smokers	Sequencing	Region if 16S	Citation
Boaden, 2017	Saliva, buccal mucosa, tongue, gingiva and hard palate	Describe the bacterial profile of the oral flora during the first two weeks following a stroke, examining changes in the condition of the oral cavity and infections	Patients with stroke (n = 50)	20% current, 30% prior-smokers	16S	V1–V9	[75]
Fåk et al., 2015	Whole mouth swab	Elucidate whether the oral microbiota composition differed between patients with asymptomatic and symptomatic atherosclerosis	Asymptomatic atherosclerosis (n = 35), symptomatic atherosclerosis (n = 27), control (n = 30), total (n = 92)	Current smokers: 9% asymptomatic atherosclerosis, 23% symptomatic atherosclerosis, 21% control	16S	V1–V2	[70]
Gordon et al., 2019	Subgingival plaque	Characterize and compare the oral microbiome between four study groups based on BP status in postmenopausal women	Normal BP (n = 179), stage I hypertension (n = 106), Stage II hypertension (n = 42), patients on hypertension medications, irregardless of BP (n = 119), total (n = 446)	Current 4.5%, former 39.7% normal BP; current 2.8%, former 47.2% stage I; current 2.4%, former 40.5% Stage II, current 2.5%, former 45.4% patients on hypertension medications	16S	V3–V4	[69]
Leskelä et al., 2020	Saliva	Investigate associations of specific oral bacteria and LPS neutralizing capacity in a case–control study of ischemic stroke	Controls (n = 100), Patients with stroke (n = 98), total (n = 198)	Current smokers: 12% controls, 28% patients with stroke	Targeted qPCR sequencing	N/A	[63]
Liljestrand et al., 2018	Subgingival plaque	Study the association between periodontal pathogen burden, saliva and serum LPS activity and how periodontitis and coronary artery disease interrelate with them	Control (n = 123), stable CAD (n = 184), ACS (n = 169), ACS-like, no CAD (n = 29); total (n = 505)	Ever smokers: 46.3% control, stable CAD, ACS, ACS-like, no CAD; 52.9% total	DNA hybridization	N/A	[64]
Kannosh et al., 2018	Subgingival plaque	Assess temporal changes in the frequency of periodontal bacteria in the subgingival plaque and in atherosclerotic blood vessels of patients with atherosclerosis	Patients with atherosclerosis (n = 100)	Smokers: 55% of total sample	Targeted 16S sequencing	N/A	[67]
Koren et al., 2011	Oral cavity swab	Aimed to address: Is there a core atherosclerotic plaque microbiota? Are bacteria present in the plaque also detectable in the oral cavities or guts of the same individuals? Is an altered oral or fecal microbiota associated with atherosclerosis?	Controls (n = 15), patients with atherosclerosis (n = 15); total (n = 30)	Current smoker: 0%, controls, 40% patients with atherosclerosis	16S	V1–V2	[74]
Mahalakshmi et al., 2017	Subgingival plaque	Determine the incidence of anaerobic periodontopathic bacterial co-occurrences in periodontitis and atherosclerosis	Control patients without periodontitis or systemic disease (n = 100), patients with atherosclerosis (n = 65); patients with periodontitis but no systemic disease (n = 59); total (n = 224)	Current or past smokers were excluded from the study	Targeted 16S sequencing	N/A	[71]
Nikolaeva et al., 2019	Subgingival plaque	Characterize composition of subgingival biofilm with periodontopathogenic bacteria species and endothelium-dependent vasodilation in patients with chest pain and concomitant periodontitis	Patients with angina pectoris (n = 15); patients with acute myocardial infarction (n = 15); Patients with chest pain but no coronary artery disease (n = 15); total (n = 45)	Current smokers: 80% patients with angina pectoris; 60% patients with acute myocardial infarction; 47% patients with chest pain but no coronary artery disease	Targeted 16S sequencing	N/A	[72]
Perry et al., 2020	Saliva	Establish how oral bacteria are related to cough sensitivity and pneumonia in a clinical stroke population	Patients with atherosclerosis (n = 100)	Not reported	Targeted qPCR	N/A	[76]
Serra e Silva Filho et al., 2014)	Subgingival plaque	Assess microbial diversity of the subgingival environment and atheroma plaques of patients with periodontitis and obstructive coronary artery atherosclerosis	Patients with periodontitis and atherosclerosis (n = 18)	Smokers: 55.6% of total sample	16S	V1–V9 (27f/ 1492r primers)	[68]
Su et al., 2019	Tongue dorsum	Study the association between oral bacteria on the tongue dorsum and factors associated with oral health and systemic disease in middle-aged and elderly patients	Total sample (n = 70)	Smokers: 11% of total sample	Targeted PCR sequencing	N/A	[73]
Ziebolz, Jahn, et al., 2018	Subgingival plaque	Detect periodontal pathogens DNA in atrial and myocardial tissue, and to investigate periodontal status and their connection to cardiac tissue inflammation	Patients undergoing surgery for aortic valve stenosis (n = 30)	Smoking history was present in only 8 patients: average of 27.2 ± 21.8 pack/years in that subset	Targeted PCR sequencing	N/A	[66]
Ziebolz, Rost, et al., 2018	Subgingival plaque	Detect correlations of microbiological DNA, inflammatory proteins, and infection parameters in patients with periodontal disease and valvular heart disease	Patients undergoing surgery for aortic valve stenosis (n = 10)	Smokers: 30% of total sample	Targeted PCR sequencing	N/A	[65]

*16S* 16S ribosomal ribonucleic acid sequencing, *ACS* Acute Coronary Syndrome, *BP* blood pressure, *CAD* Coronary Artery Disease, *DNA* deoxyribonucleic acid, *LPS* lipopolysaccharide, *PCR* polymerase chain reaction, *qPCR* quantitative polymerase chain reaction, *V* variable region of the 16S ribosomal ribonucleic acid gene

The RA of *Aggregatibacter*, *Campylobacter*, *Fusobacterium*, and *Porphyromonas* were associated with CVD in several studies (Fig. 1B). For example, increased RA of *A. actinomycetemcomitans* in saliva samples was seen in patients with ischemic stroke [63], increased RA of *A. actinomycetemcomitans* in periodontal pocket/subgingival samples occurred in patients with coronary artery disease [64], acute coronary symptom [64], and valvular heart disease [65, 66], and *A. actinomycetemcomitans* was present in both subgingival plaque and samples of coronary artery atherosclerotic samples [67]. Increased RA of *Campylobacter rectus* in periodontal pocket/subgingival samples also occurred in patients with coronary artery disease [64], acute coronary symptom [64], and valvular heart disease [65–67], and *C. rectus* was present in both subgingival plaque and samples of coronary artery atherosclerotic samples [68]. *Campylobacter concisus* was an additional species that was found in both subgingival biofilm samples and coronary artery plaques of patients with coronary artery disease [68]. Increased RA of *Fusobacterium* in subgingival samples occurred in patients with increased blood pressure [69], valvular heart disease [65, 66], and several *Fusobacterium* species were present in both subgingival plaque and samples of coronary artery atherosclerotic plaques [68]. Increased RA of *Porphyromonas* in whole mouth samples of patients with coronary artery disease [70], *Porphyromonas gingivalis* was also elevated in subgingival samples of patients with coronary heart disease [67, 68, 71, 72], valvular heart disease [65], and in smokers with a history of CVD [73]. *Porphyromonas gingivalis* was found in both subgingival and coronary artery atherosclerotic plaque samples alone and in combination with several species, including *Tannerella forsythia*, *Tannerella denticola*, *Eikenella corrodens*, and *Campylobacter rectus* [68, 71]*.*

The association between the RA of *Prevotella* in the oral microbiome and cardiovascular risk/CVD had conflicting results in the literature. There was an inverse association of the RA of *Prevotella*, *P. scopos*, and *P. intermedia* in subgingival plaque samples in patients with increased blood pressure [69] and acute myocardial infarction [72] in two studies. Other research reported a positive association of *Prevotella*, *P. shahii*, and *P. nigrescens* in whole mouth samples in patients with coronary artery disease [70], and subgingival samples in patients with increased blood pressure [69], diagnosed with hypertension [69], coronary artery disease [68, 71], valvular heart disease [65, 66], and both subgingival and coronary artery plaque samples had *P. nigrescens*, *P. intermedia*, and *P. loescheii* present. The RA of *Treponema* and *Veillonella* were increased in whole mouth samples in patients with coronary artery disease [70], and subgingival plaque samples in patients with coronary artery disease [71] and diagnosed hypertension [69]. *Veillonella* was additionally identified in both whole mouth samples and in carotid artery atherosclerotic plaque samples [74].

Increased blood pressure was associated with a reduced RA of *Actinomyces* and increased RA of *Selenomonas* in subgingival plaque samples [69], respectively, and both *Actinomyces* and *Selenomonas* were preset both in subgingival, and coronary artery atherosclerotic plaque samples [68]. *Capnocytophaga leadbetteri* and *Eikenella corrodens* were found in both subgingival plaque and coronary artery atherosclerotic plaque samples [68], and an increased RA of these bacteria in subgingival biofilm samples occurred in patients with valvular heart disease [65, 66]. The RA of *Streptococcus* and several *Streptococcus* species were decreased in saliva and oral samples of patients with elevated blood pressure and ischemic stroke compared to patients without CVD [69, 75, 76]. Finally, species-level *Filifactor alocis* and *Treponema denticola* were seen in both subgingival and coronary artery plaque samples in patients with coronary artery disease [67, 68, 71]. See Additional file 3: Table S2 for all cardiovascular-associated oral bacteria responses in the reviewed studies.

## Oral microbiome as an intersection between smoking and CVD: potential/plausible mechanisms

Smoking remains a significant risk factor for CVD development, and differences in the bacterial community of different oral niches have been associated with both smoking and CVD in the studies outlined in this review. The association between smoking and alpha diversity differed across studies, with some studies reporting an increase in alpha diversity, others reporting a decrease and others reporting no association of alpha diversity with smoking. Furthermore, many research studies did not report alpha diversity associations and focused solely on differential abundance differences in specific bacteria of the oral microbiome. Nevertheless, differences in alpha diversity associations with cigarette smoking may be related to different oral microbiome sampling sites, studies not powered to evaluate smokers versus non-smokers or different confounders present that may influence the overall bacterial community. Future research studies using consistent alpha diversity metrics will allow continued cross-study comparison across oral microbiome sampling sites and will inform the relationships between smoking and alpha diversity.

**Fig. 2:**
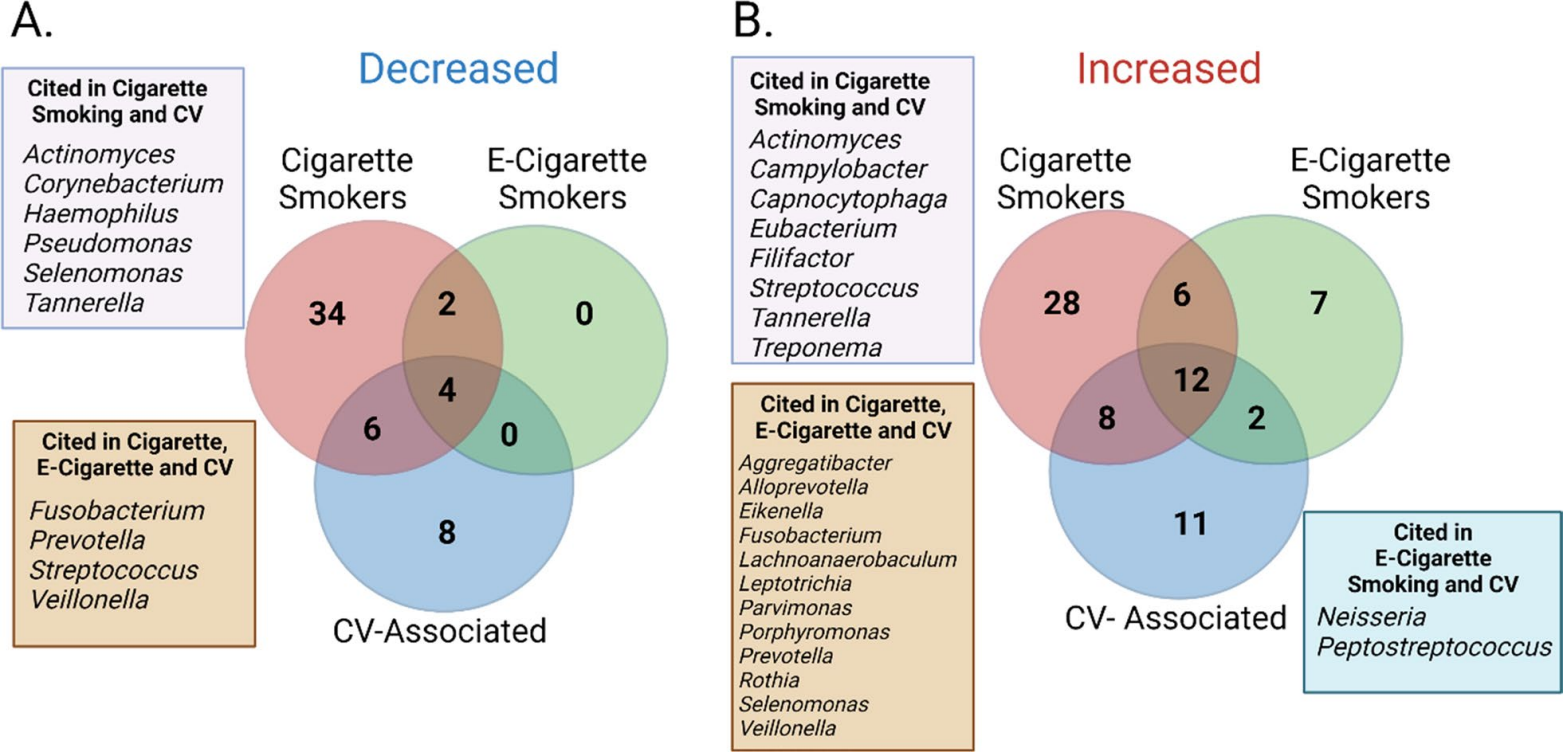
**A** Shared and unique oral bacterial taxa that are decreased in association with cigarette smoking, e-cigarette smoking and CV-associated risk or disease. **B** Shared and unique oral bacterial taxa that are increased in association with cigarette smoking, e-cigarette smoking and CV-associated risk or disease. Bacteria summary tables from Additional file 2: Table S1 (oral microbiome and smoking) and Additional file 3: Table S2 (oral microbiome and cardiovascular risk/disease) were imported into JMP™ Version 14 Data Discovery Statistical Software, and species-level bacteria was collapsed to the genus level. If a genus-level bacterium was mentioned as increased or decreased in association with cigarette smoking, e-cigarette smoking or CVD, it was binned in the respective category. See Additional file 4: Table S3 for a summary of shared and unique bacteria. *CV* cardiovascular, *e-cigarette* electronic cigarette. Figure created with BioRender.com

**Fig. 3:**
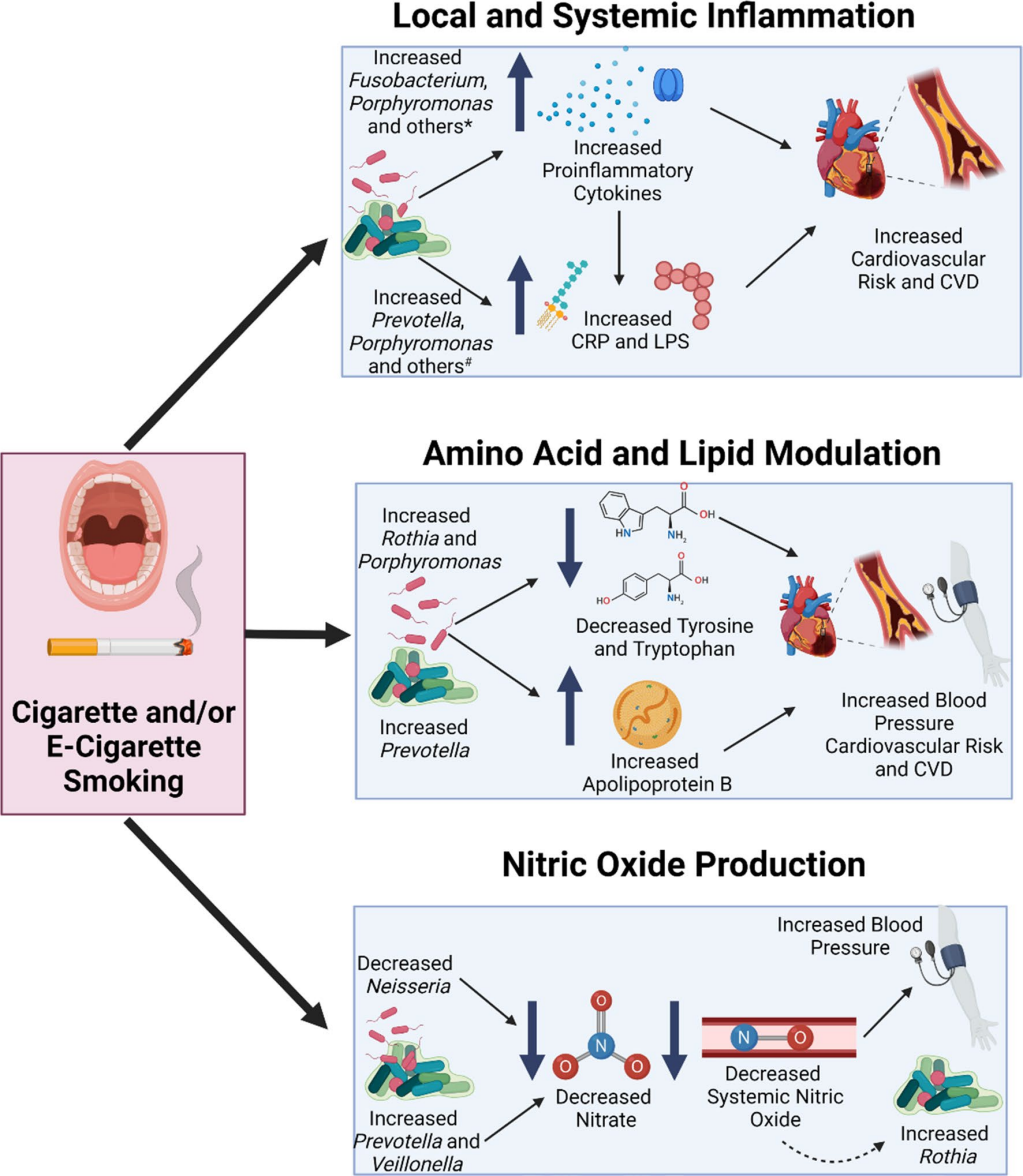
Schematic summarizing hypothesized pathways where the oral microbiome mediates cardiovascular risk and disease in response to smoking. **A** An increased RA of multiple bacteria associated with smoking including *Fusobacterium*, *Porphyromonas*, *Lachnoanaerobaculum Parvimonas*, *Mogibacterium Streptococcus*, *Selenomonas*, and *Rothia* in the oral microbiome have been positively associated with increased proinflammatory cytokine levels. A smoking-associated increase in the RA of *Alloprevotella*, *Filifactor*, *Fusobacterium*, *Porphyromonas*, *Veillonella*, *Treponema*, and *Parvimonas* was associated with LPS levels or biosynthesis genes, while *Parvimonas* was associated with CRP. Increases in local and systemic cytokines, along with elevated CRP and LPS are associated with an increased risk of CVD. **B** Increased RA of *Rothia* and *Porphyromonas*, both elevated in the oral cavities of smokers versus non-smokers, are potentially associated with decreased tyrosine and tryptophan levels through different hypothesized mechanisms. *Prevotella* RA was increased in smokers, which is positively associated with increased Apolipoprotein B levels. Both decreases in tyrosine/tryptophan and increased Apolipoprotein B is associated with increased cardiovascular risk. **C** An increase in the oral RA of *Prevotella* and *Veillonella*, and a decrease in the RA of *Neisseria* that was documented in smokers versus non-smokers is associated with decrease nitrate levels that ultimately lead to decreased nitric oxide levels. Decreased nitic oxide levels are associated with alterations in blood pressure and CVD, and may lead to a compensatory increase in oral *Rothia* abundance in association with smoking. Figure created with BioRender.com

Through comparing the associations between oral microbial taxa and smoking (Additional file 2: Table S1) and cardiovascular disease/risk (Additional file 3: Table S2), respectively, we found oral bacteria that had shared negative (Fig. 2A) and positive (Fig. 2B) associations with smoking (both cigarette and e-cigarette) and CVD. Additionally, there were several oral microbiome-associated bacteria that were present in both subgingival samples and atherosclerotic plaques that also had positive RA associations with cigarette and e-cigarette smoking (versus non-smokers). The bacteria sequenced from atherosclerotic plaques that were also associated with smoking included *Aggregatibacter actinomycetemcomitans*, *Campylobacter rectus*, *Filifactor alocis*, *Fusobacterium*, *Leptotrichia*, *Porphyromonas*, *Prevotella*, *Selenomonas*, *Treponema/Treponema denticola*, and *Veillonella*. As these bacteria have shared associations between smoking and CVD and/or cardiovascular risk, these may be important for future studies directly examining the oral microbiome as a mediator between smoking and elevated risk of CVD. Although identifying the oral microbiome compositional changes related to smoking and CVD is important in exploratory and preliminary studies, understanding the functional roles of oral microbiome shifts will provide deeper insight into the role oral bacteria has on human pathophysiology. From our extensive review of the literature, we have identified several mechanisms in which smoking can increase CVD risk through the oral microbiome, those include (1) inflammatory mechanisms, (2) lipid and amino acid modulation, and (3) production of vasoactive metabolites (Fig. 3).

### Inflammatory mechanisms

Evidence has demonstrated that periodontal disease creates an inflammatory oral environment in which mediators may transmit to the systemic circulation. Systemic diseases such as diabetes mellitus [77, 78] and CVD [79] have also been associated with increases in oral inflammation and periodontal pathology through disease mechanisms and treatment modalities [79], suggesting a bidirectional relationship between local (oral) and systemic inflammatory processes and health. The RA of bacterial taxa associated with periodontal disease, including *Porphyromonas*, *Treponema*, *Tannerella*, *Campylobacter*, and *Prevotella* spp. were positively associated with cigarette and e-cigarette smoking in many studies (Table 3). Additionally, in studies evaluating shared oral and coronary artery plaques, atherosclerotic plaques were found to contain DNA from periodontal pathogens, indicating transmission of bacteria from the oral cavity to the heart [68, 71]. The potential link between the periodontal disease pathogens and increased cardiovascular risk or CVD has been studied extensively [32, 60, 62, 80]. Evidence has demonstrated that periodontal disease creates an inflammatory oral environment in which mediators may transmit to the systemic circulation [60].

Lipopolysaccharide (LPS): LPS, an endotoxin that is commonly found in the cell wall of gram-negative bacteria, has been demonstrated to trigger the inflammatory cascade resulting in the systemic inflammation that increases cardiovascular risk and CVD [81, 82]. There is a strong connection of periodontitis to cardiometabolic disorders, mediated by LPS, that has been reviewed in detail [83]. In the reviewed studies, LPS biosynthesis pathway genes were upregulated in subgingival plaque sample of e-cigarette smokers and tongue dorsum samples of cigarette smokers [15, 55]. Additionally, many bacteria that had positive associations with LPS biosynthesis genes had increased RA levels in the oral microbiome of cigarette and e-cigarette smokers [81, 84]. For example, *Alloprevotella tannerae* was increased in saliva samples of e-cigarette smokers [46], *Filifactor* was increased in tongue dorsum samples of cigarette smokers [41, 55], *Fusobacterium nucleatum* was increased in saliva samples of e-cigarette and cigarette smokers [46, 52, 54]; and *Prevotella* was increased in saliva, whole mouth, buccal, and tongue dorsum samples of cigarette and e-cigarette smokers [34, 38–40, 44, 46, 55, 58]*.* Other bacteria associated with LPS biosynthesis that were increased in smokers included *Porphyromonas endodontalis* in tongue dorsum and saliva samples of cigarette and e-cigarette smokers [46, 55], *Veillonella* in saliva, whole mouth, buccal, tongue dorsum, and subgingival plaque samples of cigarette and e-cigarette smokers [15, 38, 43, 44, 46, 48, 50, 55, 58], *Treponema* in saliva, whole mouth and subgingival plaque samples of cigarette smokers [34, 48, 52, 55, 59], and *Parvimonas* in saliva and subgingival plaque samples of cigarette and e-cigarette smokers [15, 46]. The RA of genus-level *Parvimonas* was also positively correlated with LPS biosynthesis genes in the oral microbiome [70]. Additionally, in one study, the LPS gene in commensal biofilm samples was upregulated 22.8-fold with nicotine-free vapor and 10.1-fold in nicotine-containing vapor in e-cigarette users versus non-smokers [15].

C-Reactive Protein, CRP: CRP is another biomarker associated with inflammation and cardiovascular risk [85]. Plasma CRP levels can reliably predict the prognosis of atherosclerosis, heart failure, and other CVD, even in otherwise asymptomatic individuals [86]. Plasma CRP levels have shown to be higher in cigarette smokers versus non-smokers in studies with [60] and without [87] inclusion of oral bacteria measures. Increased CRP transcription in the liver have been associated with increased levels of pro-inflammatory cytokines IL-6, IL-1, and TNF-α, suggesting another cardiovascular risk mechanism linked to inflammation and mediated by smoking-associated changes in the oral microbiome and elevated cytokine levels [86]. As there has been a longstanding connection between oral infection and septicemia, especially in immunocompromised patients [88, 89], oral bacterial translocation to the liver and systemic circulation through the gut is another potential mechanism that deserves future research. CRP can also indirectly activate TNF-α, reactive oxygen species, and IL-1ß in apoptotic pathways leading to a persistent systemic inflammatory state [86, 90]. *Parvimonas* was positively correlated with plasma CRP in subjects with asymptomatic and symptomatic atherosclerosis in previous research [70]. In the reviewed studies of smoking and the oral microbiome, both cigarette and e-cigarette smoking were associated with an increased RA of *Parvimonas* in subgingival plaque and saliva samples [15, 46]. Collectively, these associations provide evidence that cardiovascular risk may be mediated through cigarette and e-cigarette smoking-associated oral microbiome changes that promote a systemic inflammation driven by LPS and CRP. Future mechanistic research will elucidate the pathways underlying oral microbiome-associated LPS and CRP responses to cigarette smoking so interventional research studies can be designed to lower cardiovascular risk and CVD in these patients.

Cytokines: Oral bacteria that had significant positive associations with cigarette and e-cigarette smoking were also associated with several salivary pro-inflammatory cytokines. For example *Porphyromonas* correlated positively with proinflammatory cytokines such as IL-2, IL-13, IL-8, and IL-1ß; *Lachnoanaerobaculum* with IL-2, IL-4, IL-8, IL-13, IL-10, IL-12p70, and IFN-ɣ; *Parvimonas* and *Mogibacterium* with IL-8 and IL-1ß; and *Fusobacterium* with IL-1ß [46]. *Streptococcus* and *Rothia* (increased in several oral sites of cigarette smokers) and *Selenomonas* (increased in subgingival plaque samples of cigarette smokers) also had positive associations with pro-inflammatory cytokines IL-1ß, IL-2, IL-4, IL-6, IL-7, IL-9, IL-12 and IL-17 [91]. The RA of *Johnsonella* (increased RA in e-cigarette smokers) was positively associated with IL-1ß levels [46]. Conversely, *Peptostreptococcus* (decreased in the whole mouth and tongue dorsum samples of cigarette smokers versus non-smokers [38, 41]) was negatively associated with salivary IL-8 and IL-1ß [46]. Finally, two bacteria (*Porphyromonas gingivalis* and *Fusobacterium nucleatum*) that play pivotal roles in periodontal disease and act on macrophages, neutrophils, and monocytes to induce TNF-α, IL-6, and IL-8 production [92] were increased in smokers’ tongue dorsum [55] and saliva samples [46, 52, 54], suggesting another link between smoking, periodontal disease and inflammation-mediated cardiovascular risk. It is important to note that some of the bacteria that were positively associated with smoking and LPS biosynthesis were not periodontal pathogens but genera that are common to the healthy oral microbiome habitat (i.e. *Streptococcus* and *Veillonella*). This suggests that an imbalance of “healthy” oral-associated bacteria as a result of smoking may also contribute to increased inflammation in addition to an increased RA of periodontal pathogens. Overall, upregulation of common and unique inflammatory cytokines in salivary and gingival crevicular fluids in both e-cigarette cigarette smokers suggests that smoking alters the oral microbiome in a manner associated with increased inflammation that plausibly contributes to the increased CVD risk in this population.

### Amino acids

Increases or decreases in local (i.e. oral) and systemic amino acid levels through modulation of amino acid metabolism and biosynthesis pathways is another possible mechanism in which smoking may influence cardiovascular risk through oral microbiome-associated bacteria. Amino acids are the foundational molecules for protein catabolism and are involved in the biosynthesis of important molecules including hormones, neurotransmitters, and coenzymes. Gut microbial metabolites have been strongly associated with amino acid and lipid metabolism [93], but relationships between similar oral bacteria and associated amino acid or lipid metabolic functions is not as well known.

Tyrosine is derived from phenylalanine and is a nonessential amino acid involved in protein synthesis and signal transduction, while tryptophan is an essential amino acid also involved in the production and maintenance of enzymes and neurotransmitters [94]. In many of the evaluated studies, smoking was associated with an increase in the RA of *Rothia* in saliva, whole mouth, tongue dorsum, and buccal samples [38, 41, 44, 46, 52, 55]. Associational analyses demonstrated that oral microbiome communities with higher levels of *Rothia* were more likely to display greater utilization of tyrosine and tryptophan [55], which could lead to lower levels of these amino acids. Salivary composition changes and increased incidence of periodontitis, both associated with smoking, contribute to an oral environment where gram-positive facultative anaerobes and amino-acid degrading bacteria like *Prevotella* proliferate [95]. Similarly, smoking was associated with an increased RA of *Prevotella* across all of the oral sampling sites (Table 2). Therefore, reductions in oral amino acid levels via smoking-associated bacterial changes may occur through known mechanisms such as changes in oral environment or from other mechanisms that will be identified as more work is performed on the functional implications of oral bacteria alterations in response to e-cigarette and cigarette smoking.

It is not known if smoking-associated changes in oral amino acid levels are directly associated with plasma levels, but connections between oral disorders and decreased plasma amino acid levels have been reported [96, 97]. This topic will be of great interest to establishing connections between the oral microbiome and systemic disease as many plasma amino acids are associated with cardiovascular risk and CVD. For example, tryptophan has been thought to be inversely associated with cardiovascular risk, as serum tryptophan levels have predicted lower risk of CVD events and death related to CVD in observational studies [98, 99]. Decreased serum tyrosine levels were also reported in patients with stroke [100] and hypertension [101], and increased tyrosine intake was associated with lower peripheral and central blood pressure [102]. Low plasma serum tryptophan concentrations are associated with increased CRP and other pro-inflammatory cytokines previously discussed, subsequently increasing risk for CVD [99, 103]. Therefore, an increased understanding of pathways linking smoking to the modulation of amino acid-associated oral microbiota and the influence these bacteria have on host-microbial protein–protein interactions and systemic cardioprotective amino acids such as tyrosine or tryptophan is an exciting avenue for future research.

### Lipids

Bacteria of the oral microbiome that are impacted by smoking may also modulate cardiovascular risk through associations with lipids implicated in CVD. For example, the RA of *Streptococcus salivarius* was increased in saliva and tongue dorsum samples of cigarette smokers versus non-smokers [39, 46, 55], and was also positively associated with oleic acid biosynthesis pathways in a separate in-vitro study of cultured *S. salivarius* and metabolites [104]. Circulating levels of oleic acid, a fatty acid, are not indicative of dietary intake, and may involve hepatic synthesis by the enzyme Stearoyl-CoA desaturase-1. Interestingly, circulating levels of oleic acid were independently associated with increased rates of CVD and all-cause mortality in a large Multi-Ethnic Study of Atherosclerosis [105], and oleic acid was also positively associated with diastolic blood pressure in other research [106]. The RA of *Lactobacillus*, which was increased in whole mouth and subgingival plaque samples of cigarette smokers [50, 58], was positively associated with plasma Apolipoprotein B levels in a separate study using linear regression analysis [70]. Apolipoprotein B is being increasingly acknowledged as a main contributor to atherosclerotic CVD [107], and large cohorts studies of have found oxidized lipids like Apolipoprotein B to be associated with coronary artery disease [108]. Finally, platelet activating factor, a potent phospholipid activator, has been reported to increase in both tobacco and e-cigarette smokers through the release of free radicals, creating oxidative stress to the intercellular environment [109, 110]. The RA of *Prevotella* was found to be positively associated with platelet activating factor in an in vitro experiment in mice [111], and the RA of *Prevotella* was also increased in saliva, whole mouth, and subgingival plaque samples of cigarette smokers versus non-smokers [34, 37, 38, 40, 46, 58], suggesting a potential link between smoking, *Prevotella* and platelet activating factor. Platelet activating factor has been associated with worse stroke outcomes through its thrombotic and platelet aggregation properties in addition to promoting oxidative stress and systemic inflammation [112]. While all of these relationships point to a possibility that smoking may contribute to CVD through modulation of specific oral microbiome-associated bacteria that have relationships with amino acid and lipid levels, these studies are associational in nature. Future research confirming the above relationships and testing associations with plasma amino acid and lipid levels will inform mechanisms and pathways potentially underlying the relationship between smoking and the oral microbiome and their combined impact on cardiovascular risk.

### Nitric oxide production

Nitric oxide is a known vasodilator that decreases cardiovascular risk through reduction of blood pressure and inhibition of oxidative stress, platelet aggregation and leukocyte adhesion [113, 114]. Nitric oxide is produced endogenously by nitric oxide synthases from the amino acid l-arginine and molecular oxygen. Since human cells lack nitrate (NO_3_) reducing capability, commensal oral bacteria have been identified for their role in converting dietary NO_3_ to nitrite (NO_2_), which can be further reduced to nitric oxide [113, 115]. This alternate nitric oxide production mechanism is important for maintenance of cardiovascular integrity during periods of hypoxia. This NO_3_–NO_2_-nitric oxide entero-salivary pathway is an important pathway in which the oral cavity influences systemic physiological processes and could prove to be a potential therapeutic target. Alterations in oral bacterial communities induced by antibacterial mouthwash use, had been documented to decrease nitric oxide levels and was associated with alterations in blood pressure [116, 117]. Furthermore, studies have shown that dietary nitrate supplementation using nitrate rich foods (such as green leafy vegetables) could enhance nitric oxide production. Similar to nitric oxide, carbon monoxide is a gas that possesses vasodilatory properties, has documented interactions with the microbiome (particularly the gut microbiome), and its levels are potentially influenced by dietary consumption including fiber, leafy greens, carbohydrates and proteins [118–120]. Periodontal disease-associated alterations in oral bacteria and consequential impacts of nitric oxide and cardiovascular risk through blood pressure modulation have also been previously reviewed in the literature [17]. Nevertheless, these relationships have not been as thoroughly explored for smoking-associated modulation of oral bacteria or if other small gas production (like carbon monoxide) is modulated by smoking and/or the oral microbiome and how these associations might impact subsequent cardiovascular risk. Multidirectional interactions between vasodilatory gas and associated signaling pathways with the microbiome and host are exciting avenues for future research that potentially holds promising therapeutic benefits for cardiovascular risk and disease.

In the reviewed studies, there were significant associations between smoking and nitrate-reducing bacteria (i.e., *Rothia* and *Neisseria* [24, 121]) and bacteria that are inversely associated with nitrate and nitric oxide (*Prevotella* and *Veillonella* [24, 121]). Interestingly, the two major nitrate-reducing bacteria in the oral microbiome had disparate RA associations with smoking. For example, there was agreement across several studies that the RA of *Neisseria* was decreased in saliva, whole mouth, tongue dorsum, and subgingival plaque samples [34, 36–38, 41, 43, 47, 50, 52, 53, 55, 58], while the RA of *Rothia* increased in whole mouth, saliva, tongue dorsum and subgingival plaque samples of cigarette and e-cigarette smokers versus non-smokers [36, 38, 41, 44, 46, 52, 55, 58]. Whether the smoking-associated increase in *Rothia* RA is a compensatory mechanism in response to smoking-induced decreased nitric oxide levels resulting from oral environment changes (acidity, salivary production etc.) and reduced *Neisseria* levels or is a completely separate mechanism is unknown. Future research with direct nitric oxide measures in response to smoking-associated changes in oral microbiome RA of nitrate-reducing bacteria will continue to inform these mechanisms and pathways. Unlike associations between nitrate-reducing bacteria and smoking, there was equally as strong but consistent evidence that smoking was associated with an increased RA of bacteria that possess negative relationships with nitric oxide production and availability. For example, the RA of *Prevotella* was increased in saliva, whole mouth, tongue dorsum and buccal samples [34, 36–40, 44, 46, 52, 58], and *Veillonella* increased in saliva, whole mouth, tongue dorsum, buccal, and subgingival plaque samples of e-cigarette and cigarette smokers versus non-smokers [15, 36–38, 41, 43, 44, 46, 48, 50, 55, 58]. A previous study evaluating the RA of *Prevotella* and *Veillonella* after nitrate administration reported a decrease in both *Prevotella* and *Veillonella* after 10 days of dietary supplementation with nitrate-rich beetroot juice [122]. Nitrate supplementation also lowered blood pressure in two studies using dietary nitrate interventions [113, 123], and the one study that measured oral microbiome responses to nitrate supplementation reported greater plasma NO_2_ levels were associated with higher RA of *Rothia* and *Neisseria* and lower RA of *Prevotella* and *Veillonella* [113]. Taken together, this evidence indicates smoking-mediated responses of nitric oxide-associated oral bacteria plausibly impact cardiovascular risk, and dietary nitrate supplementation may be a way to mitigate progression of CVD through modulation of oral bacteria.

## Conclusions and future directions

This review synthesized the literature on bacteria associated with smoking and the oral microbiome, and the oral microbiome and cardiovascular risk and disease. The goal of this review was to find shared bacterial taxa between smoking and CVD, respectively, to inform future research on the role of the oral microbiome in smoking-associated cardiovascular risk and disease. We identified several shared bacteria in the oral microbiome that are associated with both smoking and cardiovascular risk, and discussed potential mechanisms that may underlie these correlations including inflammation, amino acid and lipid metabolism modulation and influence on nitric oxide production and availability. Nevertheless, some limitations of this review must be discussed. The metabolites and metabolic genes identified in the reviewed manuscripts were only measured directly or indirectly in the oral cavity. Future research studying associations between oral and plasma metabolite or cytokine levels in response to cigarette and e-cigarette smoking will continue to inform how smoking-mediated alterations in the compositional and functional profiles of the microbial environment can impact systemic physiologic processes and cardiovascular risk. Studies in our review did not provide dietary data for us to determine if diet-related byproducts or metabolites directly or indirectly impacted by smoking affect the structure and function of the oral microbiome.

Many of the studies reviewed in this manuscript used different methods for DNA extraction and sequencing, which have been known to impact microbiome results [124], and may account for some of the variability in oral microbiome bacteria association with cigarette or e-cigarette smoking in the synthesized literature. Additionally, many of the studies listed in Tables 1 and 3 used different V regions for amplicon sequencing, which may also impact annotation accuracy and the RA of oral microbiome-associated taxa [125]. Therefore, consideration of the extraction and sequencing methodology is important in the interpretation of the studies reviewed in this manuscript. Smoking was not the main outcome measure in all of the reviewed studies, and therefore not all studies may have been powered to evaluate smoking-associated oral microbiome changes. Furthermore, the percentage of current and past smokers were not reported in all of the studies that evaluated relationships between the oral microbiome and cardiovascular-associated diseases. Adequately powered studies evaluating oral microbiome and smoking associations, while controlling for previous or current smoking status in studies exploring the relationship of the oral microbiome to CVD will further identify mechanisms in which the oral microbiome mediates associations between smoking and CVD. Only two published papers included e-cigarettes in studies evaluating the impact of smoking on the oral microbiome. Although both presented a clear synopsis of multiple pathways in associations between e-cigarette smoking and both bacterial and functional responses of the oral microbiome community, they surveyed different oral microbiome habitats and therefore results could not be accurately synthesized. Future work to confirm these findings and test mechanisms and pathways will be of benefit to future advancement in the field.

Multi-modal studies linking metagenomics, metatranscriptomics and metabolomics will allow a comprehensive survey of the metabolic and functional cardiovascular implications of smoking on the oral microbiome, and will be of great interest in future research. Additionally, research integrating the multi-modal oral metagenomics data with salivary and plasma biomarkers (e.g. cytokines or cortisol) will inform some of the hypothesized inflammatory mechanisms connecting smoking-associated oral microbiome changes to CVD. Specific pathways underlying metabolite production, inflammation and communication with the host have been reported in the gut microbiome. Nevertheless, the oral environment is very different from the gut microbiome and therefore putative mechanisms surrounding gut microbiome bacteria may not necessarily replicate in the oral cavity. Therefore, future work sampling both oral and gut microbiome bacterial communities can identify shared and unique mechanistic responses to smoking and CVD, and potential implications for human health. As multiple species belong to the same taxonomic level of genus can have disparate functions in the oral microbiome, sequencing methodologies that have the ability to annotate bacteria to the species level will provide more granular taxonomic annotation and precise functional assignment.

Another important consideration in future research investigating relationships between smoking, the oral microbiome and CVD is to incorporate biobehavioral methodologies that consider environmental, health behavior and physiologic/biologic markers of health in addition to smoking such as nutritional intake, exercise, or heart rate variability. Incorporating factors that quantify health disparities and socioeconomic indicators are also needed in addition to recruiting individuals from diverse ethnicities and backgrounds in oral microbiome research, as this data is limited. An exciting potential biomarker that is emerging in the quantification of environmental and smoking-associated exposure is using epigenetic markers, such as methylation levels. For example, low methylation in the aryl-hydrocarbon receptor repressor gene at cg05575921 is strongly associated with smoking [126, 127], and may be a novel way to quantify smoking exposure and smoking-associated risk in addition to traditional measures such as subjective pack-years or cotinine levels. As the environment is a known contributor to oral microbiome composition, omitting these biobehavioral measures will prevent the field from truly advancing in the understanding of how the oral microbiome influences human disease in response to insults such as smoking across populations. Therefore, interdisciplinary teams with expertise in clinical/periodontology, microbial, nutritional and biobehavioral factors will catalyze studies powered to evaluate these relevant moderating factors, which will accelerate the field’s mechanistic understanding of relationships between smoking, the oral microbiome and CVD. Our hope is that by presenting a synthesis of shared associations between smoking and the oral microbiome, and the oral microbiome and CVD, we will stimulate future high-quality research evaluating these relationships. In the future, the potential for disease risk characterization using personalized oral microbiome strategies is feasible with increased understanding of how differences in oral microbiome communities can impact both local and systemic body systems. Interdisciplinary research incorporating cutting edge technology, paired with consideration of patient-centric factors will not only advance the current understanding of pathways linking the oral microbiome to human physiology, but also stimulate the development of non-pharmacological interventions that will mitigate or prevent the progression of cardiovascular risk to CVD and benefit human health.

## Supplementary Information

**Additional file 1:** PubMed search strategy and search terms.

**Additional file 2:** Oral microbiome bacteria, bacteria response in association with smoking, oral sampling site location and citations from reviewed studies.

**Additional file 3:** Oral microbiome bacteria, bacteria response in association with cardiovascular-associated risk measure or disease, oral sampling site location and citations from reviewed studies.

**Additional file 4:** Shared and unique responses to cigarette smoking, e-cigarette smoking and cardiovascular risk/disease stratified by increased and decreased bacterial relative abundance associations.

## Data Availability

All data generated or analyzed during this study are included in this published article and the Additional files.

## Abbreviations

CRP: C-reactive protein
CVD: Cardiovascular disease
E-cigarette: Electronic cigarette
LPS: Lipopolysaccharide
IL: Interleukin
NO_2_: Nitrite
NO_3_: Nitrate
PAF: Platelet activating factor
RA: Relative abundance
TNF: Tumor necrosis factor
