# Supplementary material for: The role of the oral microbiome in smoking-related cardiovascular risk: a review of the literature exploring mechanisms and pathways
Source: J Transl Med. 2022 Dec 12;20:584. doi: 10.1186/s12967-022-03785-x (PMC9743777; doi:10.1186/s12967-022-03785-x)
Supplement: Supplementary file 1 — Additional file 1: PubMed search strategy and search terms. [file 12967_2022_3785_MOESM1_ESM.docx]

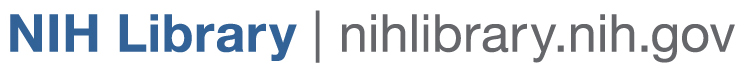


NIH Library Literature Search Request

**Completed by**: Nancy Terry, Biomedical Librarian, NIH Library | [nancy.terry@nih.gov nancy.terry@nih.gov](mailto:nancy.terry@nih.gov)

**Search(es) Requested**: oral, tongue, saliva, teeth microbiome as it relates to smoking and cardiovascular disease

**Database Searched**: PubMed

**Limits**: 10 years

**Search Originally Completed**: July 9, 2020

**Search Re-Run to Survey New Literature**: August 29, 2022

**Filters**: English

**PubMed Search Strategies**:

**1) Oral Microbiome – a)** “oral microbiome”[tiab] OR metagenomics[tiab] OR microbiome*[tiab] OR “microbial genetics”[tiab] OR “oral microbiota”[tiab] OR “mouth bacteria”[tiab] OR “mouth flora”[tiab] OR “oral bacteria”[tiab] OR “oral bacterium”[tiab] OR “oral bacterial flora”[tiab] OR “oral bacterium”[tiab] OR “oral cavity flora”[tiab] OR “oral flora”[tiab] OR “oral microbe”[tiab] OR “oral microbes”[tiab] OR “oral microbial flora”[tiab] OR “oral microbiota”[tiab] OR “oral microflora”[tiab] OR “oral microorganism”[tiab] OR “oral microorganisms”[tiab] OR “throat flora”[tiab] OR “oral microbial”[tiab] OR "oral biofilm"[tiab] OR "oral biofilms"[tiab] OR “oral bacterial profiles”[tiab] OR dysbiosis[mh] OR "oral dysbiosis"[tiab] OR “oral bacterial profiles”[tiab] OR “subgingival bacteria”[tiab] OR “supra gingival bacteria”[tiab] OR “supragingival bacteria”[tiab] OR “salivary bacterial profile”[tiab] OR “salivary bacterial profiles”[tiab] OR “oral bacterial profiles”[tiab] OR "microbial metabolite"[tiab] OR "microbial metabolites"[tiab] OR "microbial community"[tiab] OR "microbial communities"[tiab] OR biodiversity[tiab]

AND

**b)** mouth diseases/microbiology[mh]  OR mouth/microbiology[mh] OR "Mouth Mucosa"[mh] OR periodontal diseases[mh] OR saliva[mh] OR saliva*[tiab] OR salivary glands[mh] OR tongue[mh] OR tongue[tiab] OR teeth[tiab] OR oral health[mh] OR oral health[tiab] OR oral cavity[tiab] OR oral cavities[tiab] OR mouth cavity[tiab] OR mouth cavities[tiab]

**2) Smoking -** Smoking[mh] OR smoking[tiab] OR tobacco smoke pollution[mh] OR tobacco[mh] OR tobacco[tiab] OR tobacco use[mh] OR second hand smoke[tiab] OR passive smoking[tiab] OR nicotine[tiab] OR electronic nicotine delivery systems[mh] OR "electronic cigarette*"[tiab] OR "e-cigarettes"[tiab] OR nicotine[mh] OR vaping[mh] OR vaping[tiab]

**3) Cardiovascular Disease -** Heart[tiab] OR coronary[tiab] OR cardiac[tiab] OR Heart diseases[mh] OR "cardiovascular disease"[tiab] OR "cardiovascular diseases"[tiab] OR cardiovascular diseases[mh] OR atherosclerosis[mh] OR atherosclero*[tiab] OR arteriosclerosis[mh] OR "coronary artery disease"[tiab] OR "coronary artery diseases"[tiab] OR coronary disease*[tiab] OR ((oxidative stress[mh] OR "oxidative stress"[tiab]) AND (blood vessels[mh] OR vascular diseases[mh])) OR hypertension[mh] OR hypertension[tiab] OR hypertensive[tiab] OR diabetes mellitus[mh] OR diabetes[tiab] OR pre-diabet*[tiab] OR inflammation[mh:noexp] OR inflammat*[tiab] OR sympathetic nervous system[mh] OR "sympathetic nervous system"[tiab] OR "vasomotor system"[tiab]

**Search Notes**

Search strategies were combined to yield

- Smoking (#2) and the oral microbiome (#1) = 178 articles
- CVD (#3) and the oral microbiome (#1) = 509 articles
